# Artificial intelligence and environmental sustainability: a nonlinear analysis of the load capacity factor in OECD countries

**DOI:** 10.1038/s41598-026-59130-5

**Published:** 2026-08-31

**Authors:** Heng Luo, LianPing Zhang, Ying Sun, Li Zhang

**Affiliations:** 1 Jilin University of Chemical Technology, Jilin, China; 2 Jiangxi Institute of Fashion Technology, Nanchang, Jiangxi China; 3 Zhenkou Township People’s Government, Fuzhou, Jiangxi China

**Keywords:** Artificial intelligence, Environmental sustainability, nonlinear nexus, Trade openness, OECD, Environmental sciences, Environmental social sciences, Environmental studies

## Abstract

Amid the rapid expansion of artificial intelligence (AI) and growing concerns over ecological constraints, understanding the environmental consequences of emerging technologies has become increasingly important. This study investigates the nonlinear relationship between AI and environmental sustainability, which is proxied by the Load Capacity Factor (LCF), while considering the moderating effect of trade openness. Based on panel data from OECD countries between 1993 and 2023, the empirical results indicate a significant U-shaped relationship between AI and LCF. Specifically, AI initially reduces LCF but enhances environmental sustainability after exceeding a certain threshold. Moreover, trade openness is shown to flatten the U-shaped curve and shift its turning point. These findings provide meaningful policy insights for integrating digital transformation with sustainable development objectives.

## Introduction

Amidst the accelerating pace of digital transformation and growing environmental challenges, the pursuit of sustainable development has emerged as a critical global objective. Significant threats to long-term economic stability and human welfare are posed by issues such as environmental deterioration, the exhaustion of natural resources, and ecological instability. Consequently, global frameworks, including the United Nations Sustainable Development Goals, emphasize the importance of fostering technological advancement (SDG 9) while simultaneously strengthening climate action and environmental protection (SDG 13). In parallel, the global climate agenda has regained momentum, with COP30 reaffirming the international commitment to limiting global warming to 1.5 °C. Within this policy context, understanding the ecological implications of emerging artificial intelligence (AI) has become an increasingly important area of academic inquiry.

AI stands out as a pivotal technology that is fundamentally altering global economic frameworks, modes of production, and energy consumption habits. By incorporating AI into industrial processes, transportation networks, energy systems, and digital platforms, significant gains in productivity and operational effectiveness have been realized^1–3^. Nevertheless, the ecological consequences of AI adoption are not yet clearly defined. AI-powered optimization holds the potential to boost energy efficiency, refine resource distribution, and support the adoption of renewable energy^4–6^, thus promoting environmental sustainability. Conversely, the swift proliferation of AI relies on computationally intensive infrastructure, expansive data centers^7^, and a surge in digital operations, all of which could elevate energy usage and intensify environmental strain. These opposing forces imply that AI’s environmental footprint may not follow a straightforward trajectory, but rather shift throughout various phases of technological advancement.

Current empirical research^8–12^ has extensively examined the environmental impacts of AI, using indicators such as carbon emissions, ecological footprints, and energy efficiency across country-, city-, and firm-level datasets. Although these investigations yield important findings, most rely on linear modeling approaches and concentrate on particular pollution measures. Environmental sustainability, however, is a multifaceted concept. A more comprehensive metric for environmental sustainability is the Load Capacity Factor (LCF), which quantifies the relationship between human demand on nature and the biosphere’s regenerative capacity. This indicator integrates both ecological footprint and biocapacity. It shows whether economic activities exceed ecological boundaries. Despite its analytical strengths, the possible nonlinear association between the advancement of AI and environmental sustainability as gauged by the LCF has received little attention, especially within the context of advanced economies.

Recent debates highlight that AI’s environmental impact is deeply shaped by contextual factors, including the effectiveness of governance, energy mix, and the level of digital economy development. Countries leveraging low-carbon energy sources, possessing robust institutional frameworks, and advanced digital economies are better positioned to harness AI for improving energy efficiency and accelerating decarbonization^13–15^. Conversely, in economies characterized by fossil fuel dependence, weak governance capacity, or underdeveloped digital infrastructure, the expansion of AI-related activities may intensify energy demand and environmental pressure, thereby offsetting potential sustainability gains.

Simultaneously, global integration can affect the interplay between AI and environmental consequences. The degree of trade openness is a key factor that influences the spread of technological innovations, the redistribution of manufacturing processes, and the cross-border transfer of environmental costs. International trade can facilitate technology spillover effects, provide access to advanced machinery, and foster cross-border knowledge sharing^16–18^. Through these channels, trade may promote the adoption of more sustainable production methods and energy-saving technologies. On the other hand, an increase in trade volumes might also amplify the scale of industrial output and logistics operations, which could exacerbate environmental degradation^19,20^. These opposing mechanisms suggest that trade openness has the potential to modify both the direct environmental effects of technological advancement and the complex, nonlinear relationship between AI and ecological sustainability.

In developed nations, the interplay between AI and the openness of trade warrants specific consideration. Despite their high economic development, numerous OECD member states continue to grapple with significant environmental challenges. These issues primarily originate from deeply entrenched industrial frameworks and a persistent dependence on fossil fuels in critical sectors like manufacturing, transport, and energy generation^21^. Furthermore, OECD countries are responsible for a major portion of historical global emissions and have the financial resources and institutional dedication necessary to spearhead global initiatives for meeting environmental targets^22^.

Concurrently, AI is progressively reshaping production methods and international trade flows, potentially leading to intricate environmental outcomes. Technologically, AI holds the promise of improving the efficiency of resource allocation and lowering emissions via innovation. Conversely, it could also encourage increased production scales and higher energy consumption, which might negate any environmental benefits. Although these opposing effects exist, empirical research on the environmental consequences of AI in advanced economies remains limited. In particular, little attention has been paid to whether AI exhibits nonlinear environmental effects and whether trade openness moderates this relationship.

Within this framework, the present study examines the nonlinear relationship between AI and environmental sustainability, as measured by the LCF across OECD countries from 1993 to 2023. In addition to assessing the core relationship, the research evaluates how trade openness influences the observed nonlinear dynamics. The findings indicate a U-shaped relationship between AI and LCF, suggesting that AI initially undermines environmental sustainability but begins to improve it after a certain threshold is reached. Moreover, trade openness moderates the nonlinear relationship by flattening the U-curve and shifting its turning point. These outcomes provide deeper insights into the nexus between AI, trade, and environmental outcomes in advanced economies.

This study advances the literature in the following aspects. First, it advances the current understanding of AI’s role in environmental sustainability by investigating their nonlinear association, specifically through the LCF. This approach departs from the linear models and conventional metrics like carbon emissions or energy intensity frequently employed in earlier research. Second, the analysis incorporates trade openness as a moderating variable within this nonlinear AI–LCF dynamic. This offers novel perspectives on how global trade may alter the shape and strength of the connection between advancements in AI and ecological sustainability. Third, by concentrating on OECD member nations, the investigation supplies fresh empirical data from developed economies characterized by significant levels of both AI advancement and trade integration. This contributes to a more comprehensive understanding of the interplay between AI and international trade in influencing environmental sustainability.

**Fig. 1:**
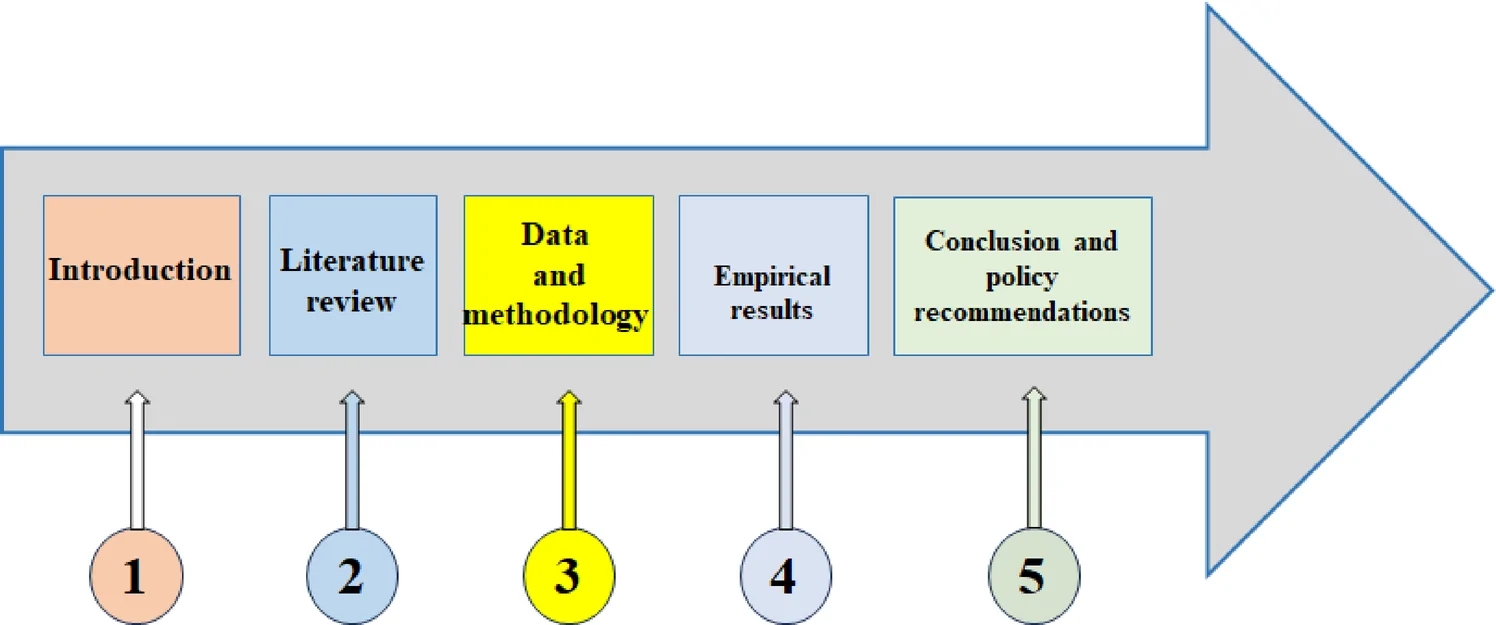
Structure of the paper. *Source*: Drawn by the author

Figure 1 illustrates the structure of the paper.

## Literature review

### Theoretical foundation

**Fig. 2:**
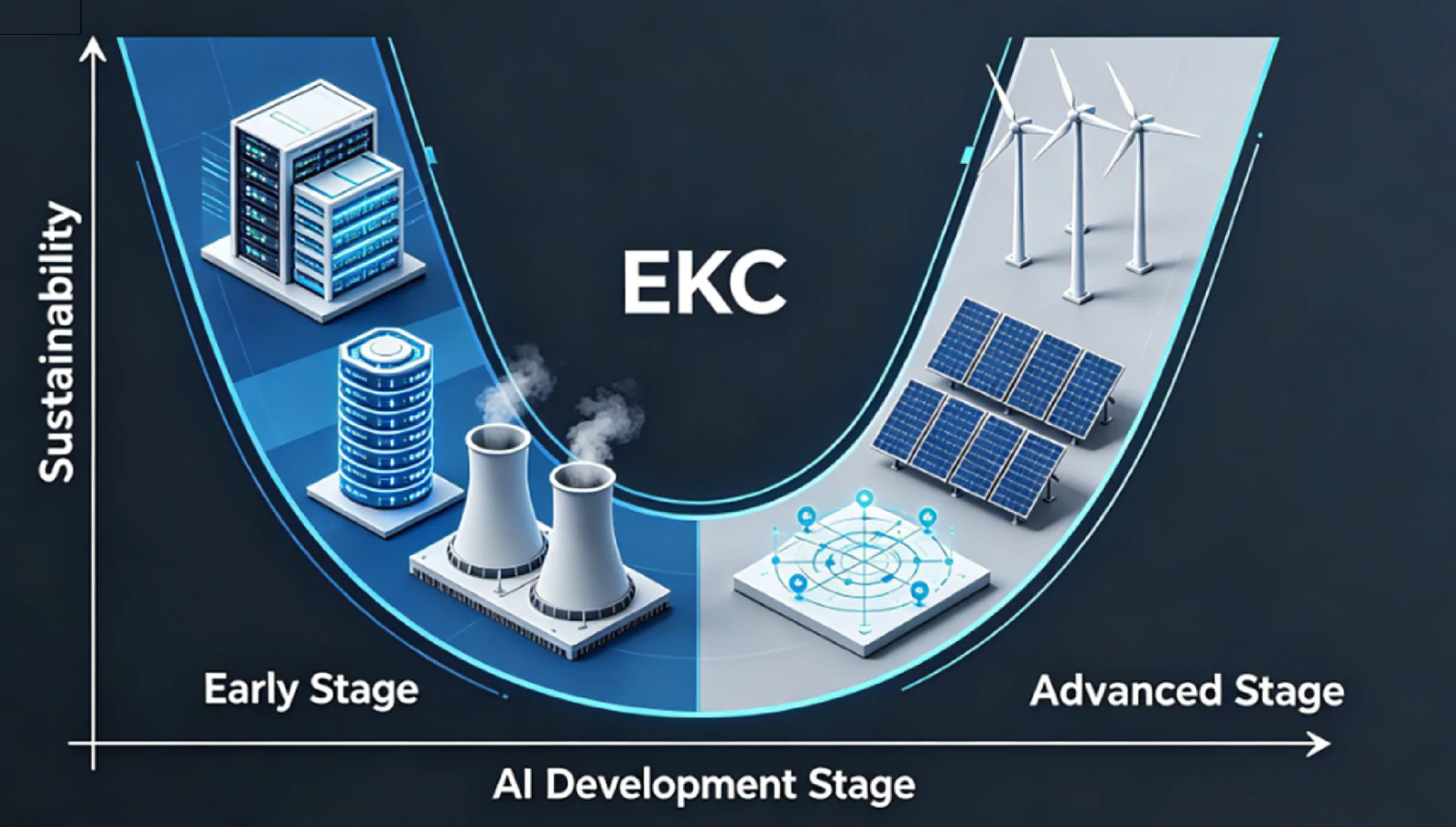
AI-environment framework incorporating EKC. *Source*: Drawn by the author

The Environmental Kuznets Curve (EKC) posits an inverted-U relationship between economic growth and environmental quality: pollution intensifies in early development stages but subsequently abates as advances in technology, shifts toward cleaner industries, and heightened environmental consciousness come into play^23^. This analytical framework can be adapted to examine AI’s role by substituting “AI” for “economic development” as the focal variable. During its initial phase, AI deployment is associated with computationally demanding training procedures and dependence on electricity generated from fossil fuels, contributing to environmental degradation^24^. However, as AI technologies advance and environmentally sustainable implementations expand, AI holds significant potential to improve energy utilization, streamline the distribution of resources, and support the seamless incorporation of renewable power sources into energy systems^10,25^. Figure 2 depicts the EKC framework.

Moreover, the Khazzoom–Brookes hypothesis offers a theoretical basis for the nonlinear rebound effect, positing that efficiency-driven economic gains are often channeled back into expansionary activities, thereby generating a rebound effect. In this context, AI will increase overall energy and resource consumption rather than reduce it.

Trade exerts influence through two interrelated effects, namely, the scale effect and the technology effect^26,27^, thus shaping the relationship between AI and the environment. Enhanced trade integration tends to expand both production and consumption volumes (scale effect), which may erode certain environmental benefits derived from AI-enhanced efficiency improvements. At the same time, international trade promotes the global dissemination of cutting-edge, resource-efficient technologies (technology effect), potentially strengthening AI’s contribution to sustainable development. Consequently, incorporating trade openness as a moderating variable enables a more differentiated analysis of how external economic linkages jointly condition AI’s environmental implications.

### AI- environment nexus

Existing studies provide mixed evidence regarding the environmental consequences of AI, which can be broadly categorized into positive, negative, nonlinear, and insignificant effects.

#### Positive evidence

According to research conducted in OECD and BRICS nations, one study^28^ reveals that AI can lower emissions in later phases by boosting energy efficiency, refining production processes and energy distribution, and supporting the smart integration of renewable energy systems. These advancements produce positive technological spillovers in green innovation, which help counterbalance the initial energy costs associated with AI implementation. Another analysis^29^, drawing on data from cities in China, establishes that AI not only cuts emissions by raising regional productivity and decreasing energy intensity but also creates notable spatial spillover effects that promote emission abatement in nearby regions. At the corporate level, research^11^ illustrates that AI aids in reducing carbon emissions by strengthening managerial and financial capabilities and improving information exchange. A comparable effect in reducing SO_2_ emissions is documented in a study of Chinese companies^30^. Corresponding carbon footprint reduction effects are also documented in^31^ for Asian developing economies^32^, for leading AI-driven industrial nations, and^33^ across 16 emerging countries.

#### Negative effects

In contrast^34^, contends that AI actually contributes to higher emissions within global fisheries. This is because existing AI applications depend heavily on energy-consuming computing systems and promote an increase in overall production scale. Consequently, the environmental impacts associated with digital adoption outweigh any possible efficiency benefits. Similarly, research by^7^ indicates that within OECD nations, AI contributes to a growth in carbon emissions. The creation and operation of AI technologies are heavily reliant on energy-demanding computing hardware and electricity generated from fossil fuels. Meanwhile, the anticipated long-term advantages for efficiency and sustainability have not yet been fully realized, leading to a net increase in emissions in the short term. Further supporting this, a study covering 54 countries by^25^ reveals that while AI offers significant potential for driving technological and economic change, its current deployment and developmental patterns come with considerable environmental drawbacks. These factors ultimately lead to an overall rise in emissions.

#### Nonlinear evidence

Nonlinear relationships are often examined by incorporating quadratic terms. For instance, drawing on a dataset encompassing 81 nations, one study^9^ demonstrates that the environmental consequences of AI are phase-specific. In the initial stages, AI adoption is associated with heightened carbon emissions and energy intensity, alongside a suppression of renewable energy uptake. However, as the technology advances, it eventually produces beneficial green spillovers, which alleviate environmental strain and encourage the growth of renewable energy. This pattern of an initial increase followed by a decrease is similarly identified in Chinese cities, where research^35^ observes an inverted U-shaped curve linking AI innovation and carbon emissions. Further supporting this staged effect, another analysis^36^ of Chinese cities confirms that AI initially worsens carbon emissions and energy intensity while hindering renewable energy use, but ultimately transitions to generating positive environmental spillovers as it matures. A study by^37^, based on data from 60 countries worldwide, also finds an inverted U-shaped relationship between AI and carbon emissions.

Alternatively, some analyses utilize threshold regression models to capture nonlinear patterns. For instance, the study by^12^ posits that AI strengthens sustainable development capacity, with a discernible threshold effect when AI is the threshold variable in Chinese cities. Once the threshold is crossed, the coefficient for AI diminishes yet remains positive.

#### Insignificant evidence

One study^38^ reports no statistically significant association between AI adoption and environmental sustainability in France. The authors argue that realizing AI’s ecological advantages likely hinges on the presence of enabling factors, such as supportive regulatory frameworks, cross-sectoral implementation, technology-driven knowledge diffusion, and sufficient investment in digital and green infrastructure, many of which remain underdeveloped in the French context.

Table 1 summarizes the AI-environment-related studies.

**Table 1:** AI-environment-related studies. *Source*: Compiled by the author

References	Sample	Explained variable/explanatory variable	Nexus
^28^	OECD and BRICS nations	AI/ emissions	−
^29^	Cities in China	AI/ emissions	−
^11^	Chinese A-share listed companies	AI/ carbon emissions	−
^30^	Chinese companies	AI/ SO_2_ emissions	−
^31^	Asian developing economies	AI/ carbon footprint	−
^32^	Leading AI-driven industrial nations	AI/ ecological footprint	−
^33^	16 emerging countries	AI/ ecological footprint	−
^34^	Global fisheries	AI/ emissions	+
^7^	OECD nations	AI/ carbon emissions	+
^25^	54 countries	AI/ CO_2_ emissions	+
^9^	81 nations	AI/ carbon emissions	Inverted U-shaped
^35^	Chinese cities	AI/ carbon emissions	Inverted U-shaped
^37^	60 countries	AI/ carbon emissions	Inverted U-shaped
^12^	Chinese cities	AI/Urban sustainable development capacity	Threshold
^38^	France	AI/biocapacity and ecological footprint per capita	Not significant

### Trade- environment nexus

The effect of trade openness on sustainability remains widely debated in the literature. For example, research on the BRICST nations by^20^ highlights that trade diversification raises environmental standards by increasing the share of environmentally sustainable goods in exports and reducing dependence on non-renewable energy. Similarly, research findings from^39^ highlight that in countries like Kenya and Algeria, trade openness is associated with reduced CO_2_ emissions, corroborating the pollution halo hypothesis. According to this hypothesis, trade with developed economies promotes technological diffusion, whereby knowledge transfer from industrialized nations enables developing countries to adopt more environmentally sustainable production methods. Studies by^40,41^ further elaborate on the technological externalities of trade, arguing that imports of advanced technologies facilitate the implementation of energy-saving strategies, and renewable energy, thereby mitigating environmental degradation.

Conversely, some studies report conflicting evidence on the environmental consequences of trade. According to^42^, trade liberalization could lead to increased emissions in certain economies, such as Saudi Arabia—a trend that may be attributed to a dominant scale effect outweighing technique-based improvements. In such cases, greater integration into global trade can expand industrial production and raise energy consumption linked to trade activities, thereby exacerbating emission levels.

### Gap

Research has increasingly examined how AI affects the environment from national, municipal, and corporate perspectives. However, the nonlinear relationship between AI and environmental sustainability, especially when evaluated using the LCF, remains largely unexplored. Most existing studies rely on linear model specifications or focus on alternative environmental indicators, such as carbon emissions and energy intensity. Furthermore, although trade openness has been widely studied for its direct environmental effects, its potential role in influencing nonlinear dynamics has received limited scholarly attention. In light of the preceding analysis, this research advances the following:

#### H1

*A curvilinear relationship exists between AI and the LCF.*

#### H2

*Trade openness moderates the curvilinear relationship between AI and the LCF.*

## Methodology

### Data

This research assembles a balanced panel dataset by combining country-level statistics from a range of authoritative international databases. The objective is to investigate the relationship between AI and environmental sustainability over the period 1993 to 2023. The LCF, which serves as the dependent variable, is calculated using data from the Global Footprint Network (GFN)(https://www.footprintnetwork.org/). The primary independent variable, representing AI, is quantified by the adoption of industrial robots, with figures sourced from the International Federation of Robotics (https://ifr.org/).

Diverging from traditional studies that often use emission-based metrics like carbon emissions or total greenhouse gas emissions to gauge environmental quality, this paper employs the LCF as a more comprehensive measure of ecological health. The LCF is calculated as the quotient of a nation’s biocapacity divided by its ecological footprint. This ratio illustrates the equilibrium between the availability of ecological resources and the consumption demands of the human population. An LCF value greater than one signifies that ecological supply exceeds demand, denoting a state of environmental sustainability. In contrast, a value below one indicates an ecological deficit and points to environmental strain.

The advantages of the LCF framework are derived from multiple factors. Firstly, it integrates a variety of ecological aspects, such as arable land, pastureland, timber resources, fisheries, developed areas, and carbon absorption potential, thereby facilitating a multi-faceted analysis of ecological performance. Secondly, LCF incorporates both the supply of ecological assets and the pressures from consumption, offering a viewpoint centered on long-range sustainability instead of a narrow emphasis on short-term emission patterns. Thirdly, the LCF quantifies the alignment between humanity’s ecological demand and the biosphere’s ability to regenerate, thereby shifting focus from mere environmental stress, such as ecological footprint and CO_2_ emissions, to systemic sustainability. Therefore, the application of LCF in this research permits an evaluation of the environmental effects resulting from advances in AI from a more holistic and sustainability-focused standpoint.

Following the approach in^43^, artificial intelligence is quantified as the natural logarithm of (1 + the stock of industrial robots). The adoption of industrial robots acts as a significant proxy for AI development, given that robotics represents a primary domain of AI implementation. The growing integration of these automated systems underscores both the expanding adoption and the continuous advancement of AI within the automation sector^44,45^.

Considering that the LCF may be influenced by a range of structural and macroeconomic factors, this analysis incorporates several additional control variables sourced from World Development Indicators (WDI) (https://databank.worldbank.org/source/world-development-indicators/) to mitigate potential bias arising from omitted factors.

Financial development (FD) is considered a key factor influencing environmental sustainability, measured by domestic credit to the private sector as a share of GDP. The influence of financial development on the LCF may manifest through multiple pathways. A mature financial sector can facilitate investment in green technologies, renewable energy, and environmentally sustainable infrastructure^46,47^, potentially enhancing biocapacity and improving the efficiency of resource utilization in relation to ecological demands. On the other hand, financial deepening may also stimulate industrial expansion, elevate consumption levels, and increase energy use^48,49^, which could lead to a larger ecological footprint and a reduction in the LCF. Therefore, the net effect of financial development on the LCF remains theoretically ambiguous and requires empirical investigation.

Second, the analysis incorporates natural resource rents (NRR) to account for the role of resource endowments. These rents are calculated as total natural resource rents expressed as a percentage of GDP. Abundant resources may strengthen a country’s fiscal capacity^50,51^, allowing for higher environmental expenditures and potentially raising the LCF. On the other hand, the resource curse hypothesis posits that excessive dependence on natural resources can result in environmental degradation, overexploitation, and carbon-intensive growth trajectories^52,53^, thereby increasing ecological pressure and diminishing the LCF.

Finally, the model accounts for economic growth by incorporating the natural logarithm of GDP per capita. On the one hand, economic activity often relies on resource-intensive sectors and fossil fuels, which can increase the ecological footprint and reduce the LCF^54–56^. On the other hand, structural transformation, technological advancement, and greater environmental awareness may promote cleaner production and more efficient resource use^57^, thereby enhancing the LCF.

To examine the moderating effect of trade openness (Trade), this study employs the ratio of total trade to GDP as the indicator.

Data for both the moderating variable and the control variables are obtained from the WDI database.

The measurements, and data sources of all variables, along with their descriptive statistics, are summarized in Table 2.

**Table 2:** Baseline outcomes. *Source*: Compiled by the author

Variable	Measurements	Source	*N*	Mean	Sd	Min	Max
LCF	Biocapacity/ecological footprint	GFN	961	0.741	0.609	0.106	2.578
AI	Industrial robots stock	IFR	961	8.383	3.948	0	14.36
FD	Private sector credit/GDP	WDI	961	95.72	47.45	20.48	184.8
NRR	Total natural resource rents/GDP	WDI	961	1.184	1.753	0.0190	6.514
GDP	GDP per capita	WDI	961	10.19	0.689	8.404	11.51
Trade	Trade activities/GDP	WDI	961	83.44	40.99	15.72	250.1

### Methodology

To examine the relationship between AI and the LCF and the moderating effect of Trade, this study employs a two-way fixed effects model. This methodology accounts for unobserved time-invariant country-specific heterogeneity and common time-specific shocks, thereby mitigating omitted variable bias and enhancing the reliability of the coefficient estimates^58,59^. Hypothesis 1 is tested using Eq. (1), in which the LCF stands for the load capacity factor and AI denotes artificial intelligence. In line with^37,60^, this study incorporates the squared term of the explanatory variable to account for potential nonlinear relationships. CC represents a set of control variables. Country-specific fixed effects are captured by u_i_, time fixed effects by ν_t_, and the stochastic error term by ε_it_.

1 $${\mathrm{LCF}}_{{{\mathrm{it}}}} = \upalpha _{0} + \upalpha _{1} {\mathrm{AI}}_{{{\mathrm{it}}}} + \upalpha _{{\mathrm{2}}} {\mathrm{AI}}_{{{\mathrm{it}}}}^{2} + \sum\limits_{{{\mathrm{a}} = 1}}^{3} {\upbeta _{{\mathrm{a}}} } {\mathrm{CC}}_{{{\mathrm{it}}}} + {\mathrm{u}}_{{\mathrm{i}}} + {\mathrm{v}}_{{\mathrm{t}}} + \upvarepsilon _{{{\mathrm{it}}}}$$

To test Hypothesis 2, Eq. (2) introduces interaction terms to examine the hypothesized moderating effect, where Trade represents the level of trade openness. All other variables are consistent with those in Eq. (1).

2 $${\mathrm{LCF}}_{{{\mathrm{it}}}} = \upalpha _{0} + \upalpha _{1} {\mathrm{AI}}_{{{\mathrm{it}}}} + \upalpha 2{\mathrm{AI}}_{{{\mathrm{it}}}}^{2} + \upalpha _{3} {\mathrm{AI}}_{{{\mathrm{it}}}} *{\mathrm{Trade}}_{{{\mathrm{it}}}} + \upalpha _{4} {\mathrm{AI}}_{{{\mathrm{it}}}}^{2} *{\mathrm{Trade}}_{{{\mathrm{it}}}} + \upalpha _{5} {\mathrm{Trade}}_{{{\mathrm{it}}}} + \sum\limits_{{{\mathrm{a}} = 1}}^{3} {\upbeta _{a} } {\mathrm{CC}}_{{{\mathrm{it}}}} + {\mathrm{u}}_{{\mathrm{i}}} + {\mathrm{v}}_{{\mathrm{t}}} + \upvarepsilon _{{it}}$$

### Pre-estimation tests

**Fig. 3:**
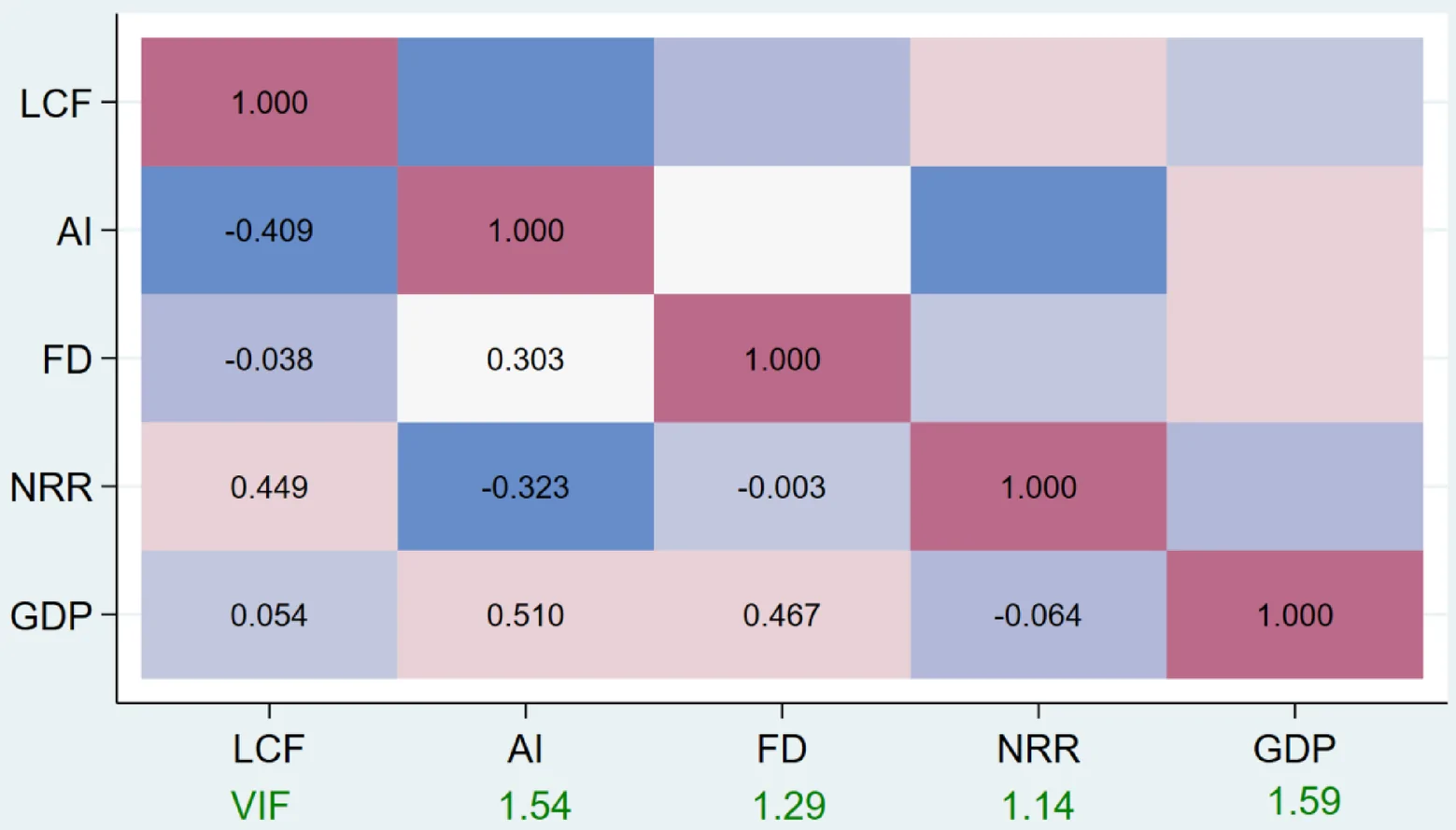
Multicollinearity diagnostics. *Source*: Generated by the author in Stata

Before conducting the regression analysis, this study performed several preliminary diagnostic tests, including multicollinearity tests, the slope heterogeneity (SH) test, the cross-sectional dependence (CD) test, and the CIPS unit root test. The corresponding results are reported in Fig. 3; Tables 3, 4 and 5.

According to Fig. 3, the correlation coefficients are all below 0.8 and the VIF values are lower than 5, indicating that multicollinearity is not a concern. The results of the SH test and CD test, reported in Tables 3 and 4, are statistically significant, suggesting the presence of slope heterogeneity and cross-sectional dependence in the panel data. Furthermore, the CIPS unit root test results presented in Table 5 show that the LCF variable is stationary at the level, while the other variables become stationary after first differencing.

**Table 3:** SH test. *Source*: Generated by the author in Stata

Test	Statistics
Δ~	31.923^a^
Δ~_adj	35.548^a^

^a^*p* < 0.01.

**Table 4:** CD tests. *Source*: Generated by the author in Stata

Test	Statistics
Pesaran CD test	24.354^a^
Pesaran scaled LM test	24.634^a^
Breusch-Pagan LM test	10548.84^a^

^a^*p* < 0.01.

**Table 5:** CIP test. *Source*: Generated by the author in Stata

Variable	LCF	AI	FD	NRR	GDP
I(0)	− 3.405^a^				
I(1)		− 3.395^a^	− 3.735^a^	− 4.877^a^	− 3.991^a^
	I(0)	I(1)	I(1)	I(1)	I(1)

^a^*p* < 0.01.

## Empirical results

To test Hypothesis 1, this study first examines the effect of AI and its squared term (AI^2^) on the LCF without including control variables. The results are shown in column (1) of Table 6. The coefficient on AI is significantly negative, while that on AI^2^ is significantly positive. Control variables are then progressively added in columns (2)–(4). Across all specifications, the coefficients for AI and AI^2^ remain consistently negative and positive, respectively, and statistically significant. Column (4) presents the benchmark specification, where the estimated coefficients for AI and AI^2^ are − 0.0334 and 0.0027, respectively, both significant. These findings suggest a nonlinear, U-shaped relationship between AI and the LCF. Thus, Hypothesis 1 is supported: before the turning point, an increase in AI is associated with a decrease in the LCF, whereas beyond the turning point, further increases in AI correspond to improvements in the LCF.

Following the method of^61^, a formal U-shaped test was conducted. As reported in Table 7, the slope at the lower bound is − 0.0334 and at the upper bound is 0.0448, both significant at the 1% level. Additionally, the U-test results lead to the rejection of the null hypothesis, thereby supporting the presence of a U-shaped relationship. The estimated turning point of 6.14 lies within the observed range of AI (0.0000 to 14.3622), confirming a statistically valid U-shaped relationship.

The U-shaped curve may reflect distinct stages in the technological lifecycle of AI. In the initial phase, the construction of essential AI infrastructure, such as data centers, high-performance computing systems, and large-scale digital platforms, generates a pronounced scaling effect^62,63^. This period of rapid expansion requires substantial electrical power, intensive computational resources, and significant material inputs for hardware manufacturing^64,65^. Consequently, the environmental impacts associated with deploying AI systems may outweigh the efficiency gains they provide, exacerbating pressure on natural resources and ecosystems and leading to an initial decline in the LCF.

As AI technologies mature and become more widely integrated across industries, their primary role shifts from infrastructure expansion to enhancing efficiency and optimizing systems^66,67^. In later stages, AI-driven solutions, including smart energy grids, intelligent transportation networks, precision manufacturing, and optimized supply chains, can significantly improve resource allocation and operational productivity^68^. These applications help reduce energy waste, refine energy management, and facilitate the integration of renewable energy into production processes, thereby promoting SDG 12 (Responsible Consumption and Production). At the same time, AI accelerates industrial transformation by promoting a transition toward knowledge-intensive and service-oriented economic sectors ^69,70^, fostering innovation and resilient infrastructure in line with SDG 9 (Industry, Innovation, and Infrastructure), while contributing to lower emissions and enhanced climate mitigation, supporting SDG 13 (Climate Action).

This finding is consistent with previous studies that report nonlinear environmental effects of AI. For instance, an inverted U-shaped relationship between AI and carbon emissions has been identified in China^36^ and across 62 countries^71^. Similarly, evidence from 56 less developed economies indicates an inverted U-shaped nexus between AI and renewable energy development^6^, while study^60^ reports an inverted U-shaped relationship between green innovation and CO_2_ mitigation in top innovative countries. However, these findings contrast with those of^33^, which finds a negative relationship between AI and ecological footprint in 16 emerging economies, and with those of^34^, which reports a positive association between AI and emissions in the global fisheries sector. This suggests that the environmental effects of AI are context-dependent, varying across countries, levels of economic development, and industrial characteristics.

Regarding the control variables, FD, NRR, and economic growth all exhibit statistically significant negative coefficients. First, the expansion of industrial activities is frequently propelled by ongoing developments within the financial sector^48,72^. This tendency exacerbates the depletion of resources and intensifies pressures on the environment, consequently diminishing the LCF. The negative sign of FD on the environment is consistent with^73^ for BRICS economies and^74^ for Belt and Road countries, but contradicts^75^ for 129 countries. Furthermore, elevated rents derived from natural resources typically signify a greater economic dependence on extractive activities. Such reliance is frequently associated with environmental deterioration and a compromised ecological carrying capacity^73^. According to research^76^, the BRICS nations also exhibit a similar detrimental effect of NRR on the environment. In contrast, a study focusing on specific Asian economies^77^ presents findings that are inconsistent with this conclusion.

Additionally, swift economic expansion can produce a scale effect that amplifies energy consumption, industrial output, and associated environmental burdens^77,78^. This is especially evident in economies where growth remains heavily tied to the use of fossil fuels and industries that are intensive in their use of resources. Collectively, these elements can adversely affect key indicators of environmental sustainability. This result is consistent with research conducted by^79^ in the context of Brazil, as well as the findings of^80^ concerning nations in the MENA region.

**Table 6:** Basic outcomes. *Source*: Generated by the author in Stata

	(1)	(2)	(3)	(4)
AI	− 0.0304^a^ (− 7.51)	− 0.0367^a^ (− 8.76)	− 0.0391^a^ (− 9.53)	− 0.0334^a^ (− 8.18)
AI^2^	0.0021^a^ (5.24)	0.0028^a^ (6.62)	0.0029^a^ (7.00)	0.0027^a^ (6.69)
FD		− 0.0006^a^ (− 5.05)	− 0.0005^a^ (− 4.16)	− 0.0008^a^ (− 6.09)
NRR			− 0.0334^a^ − 6.71)	− 0.0346^a^ (− 7.13)
GDP				− 0.1875^a^ (− 6.78)
constant	2.1308^a^ (73.68)	2.1931^a^ (70.56)	2.3314^a^ (63.57)	4.2962^a^ (14.72)
Country	YES	YES	YES	YES
Year	YES	YES	YES	YES
*N*	961	961	961	961
*R* ^2^	0.975	0.975	0.977	0.978
adj. *R*^2^	0.973	0.974	0.975	0.976

*t* statistics in parentheses.

^c^*p* < 0.1, ^b^*p* < 0.05, ^a^*p* < 0.01.

**Table 7:** The result of Inverse U shape test. *Source*: Generated by the author in Stata

	Lower bound	Upper bound
Interval	0.0000	14.3622
Slope	− 0.0334	0.0448
t-value	− 8.1804	5.3392
*P*>|t|	0.0000	0.0000
The *p*-value of overall inverse U shape = 0.0000		
Inflection point = 6.14		

### Robust test

To enhance the robustness of the baseline results, this study performs a series of supplementary analyses.

First, the core explanatory variable is substituted. Following the approach of^61,81^, AI is measured using the installation of industrial robots, which reflects the embodied application of intelligent technologies in production. The corresponding findings are shown in column (1) of Table 8.

Second, additional control variables are incorporated to address potential omitted variable bias. Expanding the set of controls helps isolate the net impact of AI on the LCF by accounting for other structural and socioeconomic drivers of environmental pressure. Specifically, urbanization is proxied by the urban population share in the total population—a measure commonly employed in the literature to capture demographic concentration and structural transformation. Urbanization may affect the LCF through various pathways. On one hand, rapid urban expansion may escalate resource consumption, energy demand, and environmental degradation, potentially lowering the LCF^82^. On the other hand, higher urbanization levels could facilitate technological diffusion, infrastructure efficiency, economies of scale in public service provision, and enhanced environmental governance^83,84^, which may in turn improve the LCF. The estimation results using the extended set of controls are reported in column (2) of Table 8.

Third, the analysis accounts for extreme global shocks. The 2008 Global Financial Crisis resulted in contractions in industrial output, trade flows, and energy demand, which may have temporarily reduced ecological pressures and influenced the LCF. Similarly, the COVID-19 pandemic from 2020 to 2021 caused substantial disruptions to economic activity, mobility, and production patterns, leading to short-term environmental impacts that could distort the estimates. To mitigate these issues, the sample excludes the year 2008 and separately omits the 2020–2021 period. The corresponding results are shown in columns (3) and (4) of Table 8.

Finally, alternative estimation methods are applied. The Driscoll–Kraay standard errors (DKSE) estimator addresses heteroskedasticity, serial correlation, and cross-sectional dependence, thereby improving inference reliability in panel data settings^85,86^. The Dynamic Common Correlated Effects (DCCE) estimator further accounts for unobserved common factors and cross-sectional dependence by including cross-sectional averages in the regression framework^87^. Results based on these alternative approaches, reported in columns (5) and (6) of Table 8, remain consistent with the baseline findings.

**Table 8:** Robustness test.

	(1)	(2)	(3)	(4)	(5)	(6)
	Replace independent	Add new	Exclude GFC	Exclude covid	DKSE	DCCE
AI		− 0.0313^a^ (− 7.70)	− 0.0337^a^ (− 8.14)	− 0.0326^a^ (− 7.66)	− 0.0334^a^ (− 3.28)	− 0.5249^b^ (− 2.28)
AI^2^		0.0024^a^ (5.98)	0.0028^a^ (6.75)	0.0027^a^ (6.17)	0.0027^a^ (3.18)	0.0245^b^ (2.10)
AIIN	− 0.0245^a^ (− 4.42)					
AIIN^2^	0.0032^a^ (5.04)					
FD	− 0.0007^a^ (− 5.40)	− 0.0007^a^ (− 5.52)	− 0.0008^a^ (− 6.20)	− 0.0008^a^ (− 5.76)	− 0.0008^a^ (− 4.53)	0.0001 (0.24)
NRR	− 0.0319^a^ (− 6.39)	− 0.0343^a^ (− 7.14)	− 0.0338^a^ (− 6.75)	− 0.0358^a^ (− 7.05)	− 0.0346^a^ (− 3.45)	− 0.0512^c^ (− 1.69)
GDP	− 0.2385^a^ (− 8.84)	− 0.1470^a^ (− 5.15)	− 0.1901^a^ (− 6.78)	− 0.1933^a^ (− 6.50)	− 0.1875^a^ (− 7.68)	− 0.0962 (− 0.72)
Urban		0.0080^a^ (4.81)				
constant	4.7698^a^ (16.47)	3.2054^a^ (8.74)	4.3288^a^ (14.61)	4.3631^a^ (13.90)	2.8651^a^ (12.95)	3.8719^b^ (2.24)
Country	YES	YES	YES	YES		
Year	YES	YES	YES	YES		
*N*	961	961	930	899	961	961
*R* ^2^	0.977	0.978	0.978	0.978		0.293
adj. *R*^2^	0.975	0.977	0.976	0.976		0.087

*t* statistics in parentheses.

^c^*p* < 0.1, ^b^*p* < 0.05, ^a^*p* < 0.01.

To address potential endogeneity issues, this research applies a two-stage least squares (2SLS) instrumental variable (IV) approach. Drawing on methodologies from prior studies^88–91^, the first lag of the explanatory variable is utilized as an instrumental variable, with its squared term also incorporated as IV2. The lagged explanatory variable meets the relevance condition, as temporal persistence leads to a strong correlation with its current value. Moreover, the exogeneity requirement is satisfied, since past values are predetermined and unlikely to be correlated with the current error term, thus maintaining the exclusion restriction. The estimation results from the IV-2SLS procedure are presented in Table 8.

As shown in Table 9, both instrumental variables (IV and IV2) exhibit significant correlations with AI and AI^2^ in the first-stage regression, which confirms the relevance of the instruments. Furthermore, the Anderson canonical correlation LM statistic and the Cragg–Donald Wald F statistic meet the respective identification test thresholds. In the second-stage estimation (column 3), the coefficients of AI and AI^2^ maintain signs consistent with the baseline regression, providing additional support for the robustness of the main findings.

**Table 9:** Robustness test. *Source*: Generated by the author in Stata

	First		Second
	(1)	(2)	(3)
	AI	AI^2^	LCF
IV1	1.0024^a^ (47.91)	0.7047^a^ (4.02)	
IV2	− 0.0051^b^ (− 2.45)	0.9248^a^ (52.44)	
AI			− 0.0297^a^ (− 7.22)
AI^2^			0.0023^a^ (5.73)
Anderson canon. corr. LM statistic	809.474 [0.0000]		
Cragg-Donald Wald F statistic	3910.575 {7.03}		
Control	YES	YES	YES
Country	YES	YES	YES
Year	YES	YES	YES
*N*	930	930	930

*t/z* statistics in parentheses.

^c^*p* < 0.1, ^b^*p* < 0.05, ^a^*p* < 0.01.

### Nonlinear moderating effects

To test Hypothesis 2, Eq. (2) is estimated by including interaction terms. As suggested by^92^, the coefficient α4 indicates whether the nonlinear relationship steepens or flattens. Specifically, a positive value of α1α4 − α2α3 implies a rightward shift of the turning point, while a negative value implies a leftward shift^61,93^.

**Fig. 4:**
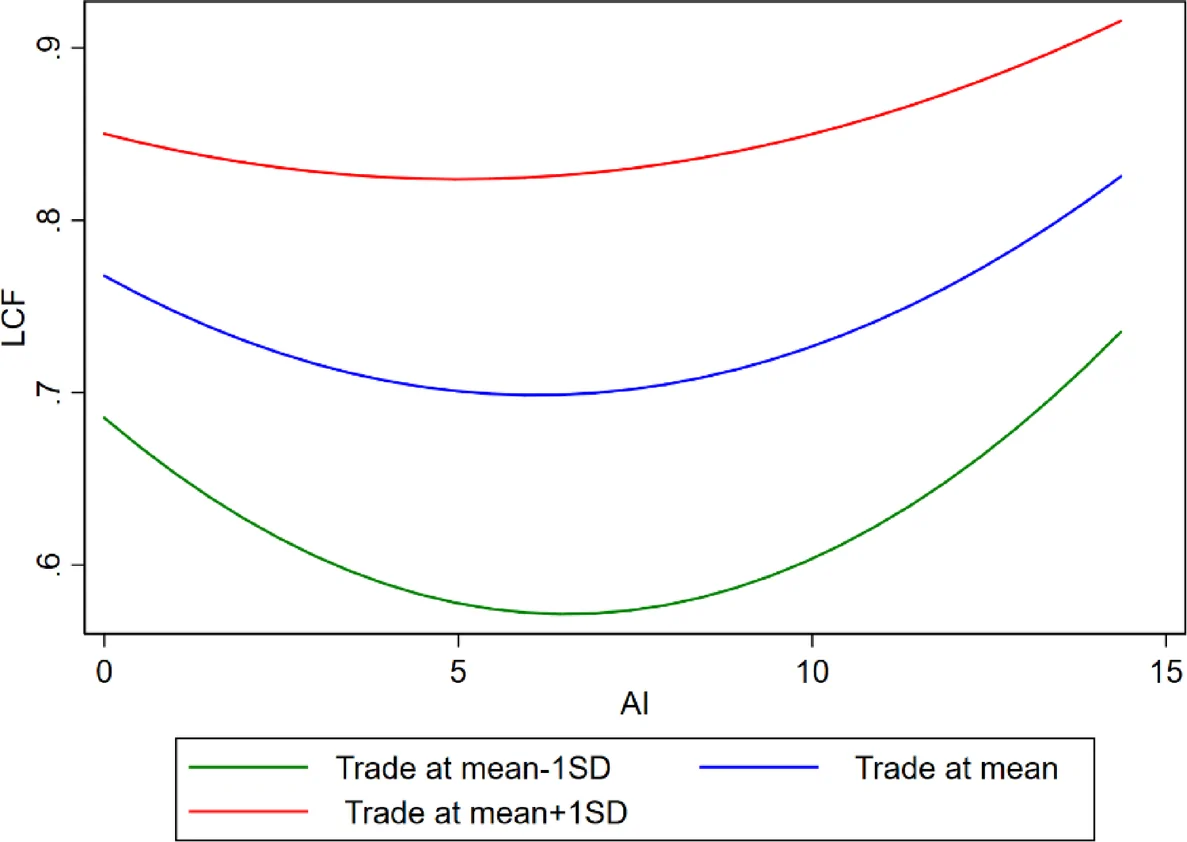
Plot of moderating effect of trade. *Source*: Generated by the author in Stata

The regression results, presented in Table 10, show that the coefficients of α3 (AI *Trade = 0.0003) and α4 (AI^2^*Trade = − 0.00002) have signs opposite to those of α1 (AI = − 0.0475) and α2 (AI^2^ = 0.0035). This suggests that trade openness reduces the curvature of the U-shaped relationship, resulting in a flatter nonlinear effect. The negative sign of α4 further indicates that greater trade openness weakens the quadratic effect of AI on the LCF, thereby smoothing the U-shaped curve. Moreover, since α1α4 − α2α3 is negative, the turning point shifts to the left, meaning that higher trade openness leads the inflection point to occur at a lower level of AI. Figure 4 illustrates the moderating effect of Trade.

On the left side of the inflection point, increased trade facilitates technological spillover by enabling the adoption and diffusion of advanced production technologies and managerial know-how^26,41,94^. This, in turn, boosts operational efficiency, strengthens environmental governance, and promotes the development of cleaner, low-carbon production methods during the early stages of AI advancement. This diminishes the adverse influence of AI on the LCF, resulting in a milder nonlinear pattern.

Beyond the turning point, AI aids in optimizing resource allocation, refining production systems, and stimulating green innovation, thus bolstering the LCF. However, when trade openness is high, nations can access cutting-edge technologies, energy-saving equipment, and sustainable products directly from global markets^26^. As a consequence, some environmental benefits that might depend on domestic AI development can instead be realized through technology imports and global specialization. This partly substitutes for AI’s contribution, lowering its marginal positive effect on the LCF and flattening the U-shaped curve.

**Table 10:** Moderating outcomes. *Source*: Generated by the author in Stata

	(1)
AI	− 0.0475^a^ (− 5.53)
AI^2^	0.0035^a^ (4.37)
FD	− 0.0003^b^ (− 2.34)
NRR	− 0.0308^a^ (− 6.65)
GDP	− 0.2931^a^ (− 10.25)
Trade	0.0020^a^ (4.17)
AI*Trade	0.0003^a^ (3.33)
AI^2^*Trade	− 0.00002^b^ (− 2.47)
constant	5.3538^a^ (17.54)
Country	YES
Year	YES
*N*	961
*R* ^2^	0.980
adj. *R*^2^	0.979

*t* statistics in parentheses.

^c^*p* < 0.1, ^b^*p* < 0.05, ^a^*p* < 0.01.

## Conclusion and implications

### Conclusion

Against the backdrop of accelerating digital transformation and mounting global climate pressures, AI has emerged as a pivotal force reshaping production structures, technological systems, and energy consumption patterns across developed economies. Drawing on panel data from OECD member countries spanning 1993 to 2023, this study investigates the nonlinear relationship between AI adoption and the LCF, while accounting for the moderating effect of trade openness. Empirical findings indicate a U-shaped relationship between AI and the LCF. Additionally, the study shows that trade openness moderates the curvature of the U-shaped relationship, leading to a flatter non-linear curve.

### Policy implications

First, it is essential for policymakers to steer the sustainable progression of AI, thereby alleviating its possible environmental burdens during the early phases of implementation. Given that the initial buildup of AI infrastructure could elevate energy requirements and resource usage, governments are encouraged to advocate for energy-efficient data centers, green computing solutions, and the incorporation of renewable energy into digital frameworks. Fostering innovation in energy-saving algorithms and smart energy management systems through research and development can accelerate the attainment of AI’s long-term ecological advantages.

Second, enhancing trade openness contributes to easing the environmental transition spurred by the uptake of AI. Liberal trade policies help disseminate cutting-edge technologies, energy-saving devices, and environmentally friendly production methods internationally. Consequently, governments ought to encourage international trade collaboration, lower import restrictions on clean technologies, and engage proactively in global technology exchanges. These steps can amplify technology spillovers, assisting nations in realizing environmental gains alongside the growth of AI-based economic endeavors.

### Future directions

First, the evaluation of artificial intelligence usage is measured at the national level using industrial robots as a proxy, which captures applied AI in production but does not reflect all forms of AI across sectors. Future research could undertake more detailed, sector-specific analyses or examine other forms of AI.

Second, the study’s sample is limited to OECD member countries. While this enhances comparability in terms of institutional and technological development, it may constrain the generalizability of the findings. The environmental effects of AI deployment in lower-income countries could differ considerably, depending on factors such as their energy composition and the robustness of regulatory systems. Expanding the research to include a broader and more varied global sample would improve the external validity of the results.

Finally, although trade openness is examined as a moderating variable from a macroeconomic perspective, the structure of trade also plays a crucial role. The environmental impacts linked to AI may vary depending on whether a country’s trade is dominated by high-technology products, energy-intensive goods, or digital services. Subsequent research could incorporate indicators of trade composition or a country’s position within global value chains to uncover more nuanced moderating mechanisms.

## Data Availability

The datasets used and/or analyzed during the current study are available from the corresponding author on reasonable request.
